# The acute phase response protein SERPINA3 is increased in tear fluid from the unaffected eyes of patients with unilateral acute anterior uveitis

**DOI:** 10.1186/s12348-021-00249-z

**Published:** 2021-07-02

**Authors:** Jon Roger Eidet, Maja Akopian, Ole K. Olstad, Øystein Kalsnes Jørstad, Morten C. Moe, Goran Petrovski, Milaim Pepaj

**Affiliations:** 1 Department of Ophthalmology, Center for Eye Research, Oslo University Hospital and University of Oslo, Oslo, Norway; 2 Department of Medical Biochemistry, Blood Cell Research Group, Section for Research, Oslo University Hospital, Oslo, Norway; 3 Department of Medical Biochemistry, Hormone Laboratory, Oslo University Hospital, Oslo, Norway

**Keywords:** Uveitis, Tear fluid, Proteomics, Biomarker

## Abstract

**Background:**

To identify candidate tear fluid biomarkers in patients with unilateral acute anterior uveitis (AAU) that can aid in the differentiation between these patients and patients with bacterial keratitis or healthy controls.

**Methods:**

Thirteen patients (40.1 ± 16.2 years of age) with unilateral AAU, seven patients with unilateral bacterial keratitis (40.2 ± 15.3 years of age), and 14 healthy subjects (41.1 ± 11.6 years of age) were included. The tear proteome of affected eyes was compared with that of the unaffected eye or healthy controls. Proteins were identified by liquid chromatography tandem mass spectrometry and enzyme-linked immunosorbent assay.

**Results:**

Relative protein ratios were detected and calculated for 272 unique proteins. Compared with healthy controls and the unaffected eye, the top upregulated proteins in AAU eyes were submaxillary gland androgen regulated protein 3B (SMR3B) and SMR3A. Similarly, the top upregulated proteins in bacterial keratitis were S100 calcium-binding protein A9 and orosomucoid 2. The acute phase response protein Serpin Family A Member 3 (SERPINA3) was increased in the healthy eye of AAU patients (*P* = 0.019) compared with healthy controls. Laser flare measurements in affected eyes of AAU patients showed positive logarithmic correlation with SERPINA3 in tear samples of the unaffected eye (*P* = 0.022). The use of SERPINA3 as a tear biomarker yielded a sensitivity of 85% and a specificity of 71% in detecting patients with AAU in the study population.

**Conclusions:**

The acute phase response protein SERPINA3 was increased in tear samples of unaffected eyes of patients with unilateral AAU compared with healthy controls. This study highlights SERPINA3 as a potential biomarker for AAU. Future research should explore the dynamic properties of SERPINA3 in the tear fluid of active and quiescent uveitis eyes.

## Background

For a patient with an acute red eye, two important diagnoses to consider are acute anterior uveitis (AAU) and bacterial keratitis. AAU and bacterial keratitis are both common and potentially sight threatening, and such patients need urgent referral to an ophthalmologist for management. Non-ophthalmologist physicians lack a test for early detection of AAU or bacterial keratitis and must rely on symptoms and plain signs, e.g. miosis, ciliary injection, or fluorescein-staining of an epithelial ulcer, which nevertheless can be subtle an easily missed. In consequence, symptoms and signs of AAU or bacterial keratitis may be misinterpreted as non-sight threatening external eye disease, delaying necessary referral to ophthalmologists.

Tear fluid is recognized as a potential source of biomarkers for a variety of diseases, both ocular and extra-ocular. Non-invasive sampling of tear fluid makes biomarkers more accessible than those of aqueous humor or vitreous. While most studies have focused on ocular surface biomarkers in dry eye or ocular allergic diseases [11, 26], biomarkers have also been proposed for intraocular diseases, such as diabetic retinopathy [17, 29]. In addition, biomarkers have been described for extra-ocular conditions, including multiple sclerosis and Parkinson’s disease [3], thereby extending the potential usefulness of tear fluid biomarkers. There are relatively few studies on tear fluid biomarkers in uveitis [4, 7, 19, 22], but novel biomarkers were recently suggested for Behcet’s disease and acute, non-infectious uveitis [18, 25]. Definite tear biomarkers for uveitis are yet to be established.

We have previously demonstrated that an inflammatory tear proteome profile involving the liver X receptor/retinoid X receptor (LXR/RXR) pathway can be detected in patients with unilateral AAU using Schirmer’s test strips [10]. In the current study, we explored the specificity of the upregulated tear proteins in unilateral AAU by comparing the tear proteome of eyes with unilateral AAU or bacterial keratitis with that of the unaffected eye and healthy controls.

## Methods

### Patients

The study took place at the Department of Ophthalmology at Oslo University Hospital, Norway. All subjects were examined by one of two trained ophthalmologists.

The AAU diagnosis was based on: 1) Symptoms: ocular pain and photophobia. 2) Signs: unilateral anterior chamber cells and ipsilateral anterior chamber flare, ciliary injection, and miosis. Exclusion criteria were: age < 18 years, pregnancy, bilateral AAU, intermediate-, posterior- or pan-uveitis, AAU secondary to suspected or known infectious disease, presence of any concurrent ocular disease (including dry eye syndrome, conjunctivitis, keratitis, eyelid disease, glaucoma, or neoplastic disease), prior severe ocular trauma, prior ocular surgery, intraocular pressure < 6 or > 21 mmHg, use of topical eye medication, use of systemic anti-inflammatory medication, or smoking.

The diagnosis of bacterial keratitis was made clinically on the basis of: 1) a corneal epithelial defect confirmed by fluorescein staining and 2) a corneal infiltrate indicative of bacterial infection. Exclusion criteria were: age < 18 years, pregnancy, presence of any ocular disease other than keratitis, prior significant ocular trauma, prior ocular surgery, ocular neoplastic disease, use of topical eye medications, use of systemic anti-inflammatory medication, or smoking.

Healthy controls were recruited among the staff at the Department of Ophthalmology. Exclusion criteria were: age < 18 years, pregnancy, use of contact lenses, any current or prior ocular disease including dry eye or ocular allergy, prior significant ocular trauma, prior ocular surgery, use of topical eye medications, use of systemic anti-inflammatory medication, or smoking.

### Laser flare meter

The degree of anterior chamber inflammation was quantified using a FM-600 laser flare meter (Kowa Company, Ltd., Tokyo, Japan). At least three valid and stable measurements were obtained per eye. Mean values, expressed in photon counts per millisecond (ph/ms), were then automatically calculated by the laser flare meter software following exclusion of outliers.

### Tear fluid sampling

We have previously described the method for using Schirmer’s strips to collect and analyze tear fluid [1, 2, 10]. Briefly, Schirmer’s test strips were placed behind the lateral third portion of the lower eyelid for 5 minutes without anesthesia and with the eye closed. Test strips were then stored at − 140 °C until analyses. In the current study, two Schirmer’s test strips were used for each eye. One strip was utilized for proteomics analysis, and the other was used for enzyme-linked immunosorbent assay (ELISA).

### Proteomics analysis

Pooled samples from the affected eye in both patient groups were compared with pooled samples from healthy controls. In addition, pooled samples from affected eyes in the two patient groups were compared with pooled samples of the unaffected eyes in the same groups. Protein extraction and analyses were performed according to protocols described previously [1, 2].

### Ingenuity pathway analysis

Lists of protein identifiers (UniProt) generated by the proteomics analysis were uploaded onto the web-delivered application Ingenuity Pathways Analysis (IPA) (QIAGEN N.V., Venlo, Netherlands) and mapped to their corresponding gene objects/molecules. The lists of gene objects in IPA were then overlaid into the Ingenuity knowledge base, which contains a global molecular network, used to identify changes in pathways and biofunctions. Thereafter, biofunction analysis in IPA identified associated pathways and biofunctions on the basis of the ratio of the number of genes in the data set that were associated with the pathway/biofunction divided by the total number of genes that were associated with the pathway/biofunction. Fischer’s Exact Test was used for statistical analysis to assess whether each pathway/biofunction was due to chance alone. The pathways/biofunctions that were represented with more molecules than expected by chance were reported.

### Enzyme-linked Immunosorbent assay

On the basis of a previous study by our group [10], current results, and potential biological relevance, the acute-phase response protein Serpin Family A Member 3 (SERPINA3) was chosen for validation with ELISA. Tear fluid proteins were extracted from the second Schirmer’s test strip, and the concentration of SERPINA3 was measured with a commercially available ELISA kit according to the manufacturer’s instructions (Human SERPINA3 – product no RAB1687; Sigma Aldrich, St. Louis, MO, United States). Protein concentration measurements of SERPINA3 were performed in singlicate (because of low sample volumes) by a microplate absorbance reader (Victor^3^; PerkinElmer, MA, United States). The concentration of SERPINA3 obtained from ELISA was normalized to total tear protein concentration. From here on the SERPINA3 concentration is expressed without its unit ([ng/ml]/total tear protein content [μg/μl]).

### Statistical analyses

Statistical analyses were performed using Statistical Package for the Social Sciences (SPSS) version 25 (IBM Corp, Armonk, NY). Comparisons of three groups were performed using one-way analysis of variance (ANOVA) with Tukey’s post-hoc test or Kruskal-Wallis H, followed by pairwise Mann-Whitney U test for parametric and non-parametric data, respectively. Comparisons of two independent groups were done using Student’s independent sample t-test for parametric data, while dependent groups were compared with Student’s paired-sample t-test for parametric data and the Wilcoxon signed-rank test for non-parametric data. Chi-square test was used for comparison of categorical data. *P*-values < 0.05 were considered significant.

## Results

### Patients

The study included 13 patients with unilateral AAU and seven patients with bacterial keratitis. Fourteen staff members at the department served as healthy controls. The study groups were age- and sex-matched and displayed comparable ethnicity (Table 1). The mean duration of symptoms prior to tear sampling was slightly higher in the AAU group than in the bacterial keratitis group, but the difference was not significant. As expected, intraocular pressure in the affected eyes of AAU patients was significantly lower than in the eyes of healthy controls. The Schirmer’s test revealed higher tear secretion in the affected eyes of AAU patients compared with the eyes of healthy controls. Also, the mean laser flare value in the affected eyes of AAU patients was significantly higher than in the eyes of healthy controls.

**Table 1:** Patient characteristics

	Healthy control	Acute anterior uveitis	Bacterial keratitis	***P***-value
N	14	13	7	–
Age (years)	41.1 ± 11.6	40.1 ± 16.2	40.2 ± 15.3	0.98^a^
Female/male	9/5	8/5	5/2	0.91^b^
Ethnicity (Caucasian/Asian/Hispanic/African)	13/1/0/0	11/1/1/0	5/0/1/1	0.38^b^
Duration of symptoms prior to tear fluid sampling (days)	N/A	4.8 ± 3.6	2.4 ± 1.5	0.13^c^
IOP in affected eye (mmHg)	14.3 ± 3.0*	10.7 ± 2.4	12.8 ± 3.2	0.008^d^
IOP in unaffected eye (mmHg)	14.3 ± 3.0*	13.8 ± 3.1	12.0 ± 2.5	0.28^a^
Schirmer’s test in affected eye (mm)	13.4 ± 8.8*	27.0 ± 12.0	16.3 ± 11.0	0.019^e^
Schirmer’s test in unaffected eye (mm)	13.4 ± 8.8*	10.8 ± 9.8	7.7 ± 1.2	0.25^f^
Laser flare meter in affected eye (ph/ms)	4.5 ± 1.1*	170.5 ± 175.7	45.5 ± 64.7	< 0.001^e^
Laser flare meter in unaffected eye (ph/ms)	4.5 ± 1.1*	4.4 ± 2.8	4.6 ± 1.3	0.98^a^
HLA-B27 positive (number of patients)	–	4^g^	–	
Bacteria identified in microbial culture (number of patients)	–	–	6^h^	

*IOP* Intraocular pressure, *Average of both eyes in healthy control; ^a^ One-way analysis of variance (ANOVA); ^b^ Chi-square; ^c^ Mann-Whitney U test; ^d^ ANOVA followed by Tukey’s post hoc test between patients with acute anterior uveitis and healthy control; ^e^ Kruskal-Wallis H test followed by pairwise Mann-Whitney U test between patients with acute anterior uveitis and healthy control; ^f^ Kruskal-Wallis H test; ^g^ one patient with ankylosing spondylitis, one patient with reactive arthritis; ^h^ S.aureus, S.epidermidis and P.acnes

### Tear fluid proteome in sick eyes versus healthy eyes

**Fig. 1:**
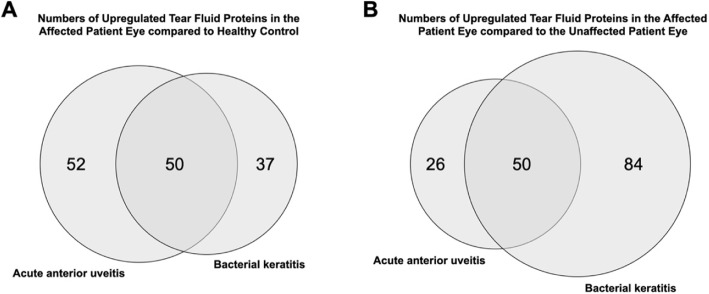
Venn diagram illustrating the number and degree of overlap for tear fluid proteins that were increased in affected eyes of patient relative to eyes of healthy controls (**A**) or unaffected eyes of patients (**B**)

For proteomics analysis, tear fluid samples were pooled from five different groups: 1) affected eyes of AAU patients, 2) unaffected eyes of AAU patients, 3) affected eyes of bacterial keratitis patients, 4) unaffected eyes of bacterial keratitis patients, and 5) eyes of healthy control. Relative protein ratios (between groups 1/2, 1/5, 3/4, and 3/5) were detected and calculated (as fold change) for a total of 272 unique proteins. Approximately one third of the tear fluid proteins that were increased in affected eyes of patients relative to unaffected eyes or eyes of healthy controls were overlapping between AAU and bacterial keratitis patients (Fig. 1).

Forty-four proteins were at least 1.5-fold increased in the affected patient eyes relative to eyes of healthy controls. When comparing the affected patient eyes with the unaffected patient eyes, 46 proteins were at least 1.5-fold increased. Table 2 summarizes the top 15 up-regulated proteins. Serum albumin concentration was decreased in tear fluid of affected patient eyes: − 1.34-fold decreased in affected eyes of AAU patients versus eyes of healthy controls; − 1.13-fold decreased in affected versus unaffected eye of AAU patients; − 1.07-fold decreased in affected eyes of bacterial keratitis patients versus eyes of healthy controls; and − 1.23-fold decreased in affected versus unaffected eyes of bacterial keratitis patients.

**Table 2:** Top 15 upregulated tear fluid protein level ratios

Affected eyes of AAU patients versus eyes of healthy controls		Affected versus unaffected eye of AAU patients		Affected eyes of bacterial keratitis patients versus eyes of healthy controls		Affected versus unaffected eyes of bacterial keratitis patients	
*Name*	*Fold change*	*Name*	*Fold change*	*Name*	*Protein*	*Name*	*Fold change*
SMR3B	8.4	SMR3A	10.7	S100A9	7.6	ORM2	14.1
SPARCL1	2.7	CNDP2	5.2	SERPINA3	3.4	CST2	5.6
PRR27	2.4	KRT4	5.0	CXCL17	3.1	CST5	4.7
ZG16B	2.3	CHI3L2	4.4	APOC3	3.1	CNDP2	3.4
EEF1A2	2.2	CST5	4.1	LXN	3.0	FGB	2.7
MUC5AC	2.1	IGHV1-69D	3.1	S100P	2.9	RARRES1	2.7
SMR3A	2.1	LTF	2.9	NDST1	2.6	CST1	2.6
LPO	2.0	KRT13	2.6	A2M	2.4	OPRPN	2.5
CALU	2.0	OPRPN	2.4	CD22	2.3	FGG	2.4
CST2	1.9	KRT19	2.3	SMR3A	2.1	FGA	2.3
SCGB2A1	1.9	SERPIND1	2.1	ZG16B	2.1	KRT19	2.3
OPRPN	1.9	APOA1	2.1	CST2	2.1	KRT13	2.2
SERPINA3	1.8	CTSD	2.0	IGKV3D-11	1.9	KRT4	2.2
MYL12B	1.7	SCGB2A1	1.9	PLA2G2A	1.9	ANXA1	2.1
ANXA3	1.6	FGG	1.9	CALU	1.8	KRT7	2.1

*AAU* acute anterior uveitis

### Ingenuity pathway analysis

The top canonical pathways identified by IPA were comparable across all groups. Notably, the top three pathways affected in the affected eyes of both AAU and bacterial keratitis patients were *acute phase response signaling*, *LXR/RXR activation,* and *FXR/RXR activation* (Table 3).

**Table 3:** Top canonical pathways

Affected eyes of AAU patients versus eyes of healthy controls	Affected versus unaffected eye of AAU patients		Affected eyes of bacterial keratitis patients versus eyes of healthy controls		Affected versus unaffected eyes of bacterial keratitis patients		
*Name*	*P-value*	*Name*	*P-value*	*Name*	*P-value*	*Name*	*P-value*
Acute phase response signaling	1.07E-26	LXR/RXR activation	2.80E-28	LXR/RXR activation	6.85E-25	LXR/RXR activation	3.20E-29
LXR/RXR activation	6.50E-26	FXR/RXR activation	8.72E-28	FXR/RXR activation	1.84E-24	FXR/RXR activation	1.05E-28
FXR/RXR activation	1.9E-25	Acute phase response signaling	1.11E-27	Acute phase response signaling	7.90E-21	Acute phase response signaling	2.05E-28
Clathrin-mediated endocytosis signaling	3.97E-12	Coagulation system	5.86E-13	Glucocorticoid receptor signaling	1.69E-11	Coagulation system	9.63E-13
Atherosclerosis signaling	4.51E-11	Glucocorticoid receptor signaling	5.61E-11	Glycolysis I	2.86E-11	Glucocorticoid receptor signaling	3.99E-11

*AAU* acute anterior uveitis

The upstream regulator analysis used by IPA to detect potential transcriptional regulators yielded similar results when comparing AAU and bacterial keratitis patients with healthy controls (Table 4). Specifically, Tumor necrosis factor (TNF), Microtubule associated protein tau (MAPT), Amyloid beta precursor protein (APP), and Presenilin 1 (PSEN1) were identified as important transcriptional regulators in both AAU and bacterial keratitis patients relative to healthy controls. When comparing affected and unaffected eyes of AAU or bacterial keratitis patients, however, there were larger discrepancies in the lists of transcriptional regulators. In particular, *lipopolysaccharide* (LPS) was identified as an important transcriptional regulator for AAU patients.

**Table 4:** Top upstream regulators

Affected eyes of AAU patients versus eyes of healthy controls		Affected versus unaffected eyes of AAU patients	Affected eyes of bacterial keratitis patients versus eyes of healthy controls			Affected versus unaffected eyes of bacterial keratitis patients	
*Name*	*p-value*	*Name*	*p-value*	*Name*	*p-value*	*Name*	*p-value*
MAPT	2.25E-26	Lipopolysaccharide	5.74E-31	MAPT	2.82E-25	MAPT	2.91E-22
APP	2.90E-23	Nitrofurantoin	2.14E-30	APP	1.69E-22	APP	6.12E-20
TNF	2.42E-18	Dexamethasone	6.35E-26	PSEN1	1.32E-18	TNF	1.82E-17
TP53	1.54E-17	Beta-estradiol	1.21E-23	TGFß1	2.21E-17	TGFß1	3.99E-17
PSEN1	2.24E-17	APP	4.26E-21	TNF	4.41E-17	IL6	1.73E-16

*AAU* acute anterior uveitis

The inflammatory profiles of the upregulated tear fluid proteins in the affected eyes of both AAU and bacterial keratitis patients were underscored by the identification of *Inflammatory response* among the top diseases and biofunctions for all compared groups (Table 5).

**Table 5:** Top diseases and bio functions - diseases and disorders

Affected eyes of AAU patients versus eyes of healthy controls		Affected versus unaffected eyes of AAU patients		Affected eyes of bacterial keratitis patients versus eyes of healthy controls		Affected versus unaffected eyes of bacterial keratitis patients	
*Name*	*p-value*	*Name*	*p-value*	*Name*	*p-value*	*Name*	*p-value*
Inflammatory response	7.17E-06 – 1.24E-45	Inflammatory response	4.76E-06 – 1.04E-45	Inflammatory response	3.46E-05 – 2.65E-42	Inflammatory response	1.28E-05 – 1.52E-46
Organismal injury and abnormalities	7.34E-06 – 1.69E-28	Neurological disease	2.76E-06 – 1.18E-35	Organismal injury and abnormalities	3.46E-05 – 2.01E-28	Organismal injury and abnormalities	1.63E-05 – 6.31E-28
Dermatological diseases and conditions	7.17E-06 – 5.17E-28	Organismal injury and abnormalities	4.76E-06 – 1.18E-35	Dermatological diseases and conditions	2.01E-05 – 3.20E-23	Dermatological diseases and conditions	9.09E-06 – 1.14E-27
Endocrine system disorders	2.44E-08 – 2.82E-26	Endocrine system disorders	1.05E-08 – 6.43E-29	Metabolic disease	2.90E-05 – 2.46E-21	Metabolic disease	2.78E-06 – 1.22E-23
Metabolic disease	3.10E-06 – 7.93E-25	Metabolic disease	3.15E-06 – 2.43E-27	Endocrine system disorders	3.07E-05 – 6.38E-20	Neurological disease	1.37E-05 – 3.58E-21

*AAU* acute anterior uveitis

### Validation of SERPINA3 in tear fluid using enzyme-linked Immunosorbent assay

**Fig. 2:**
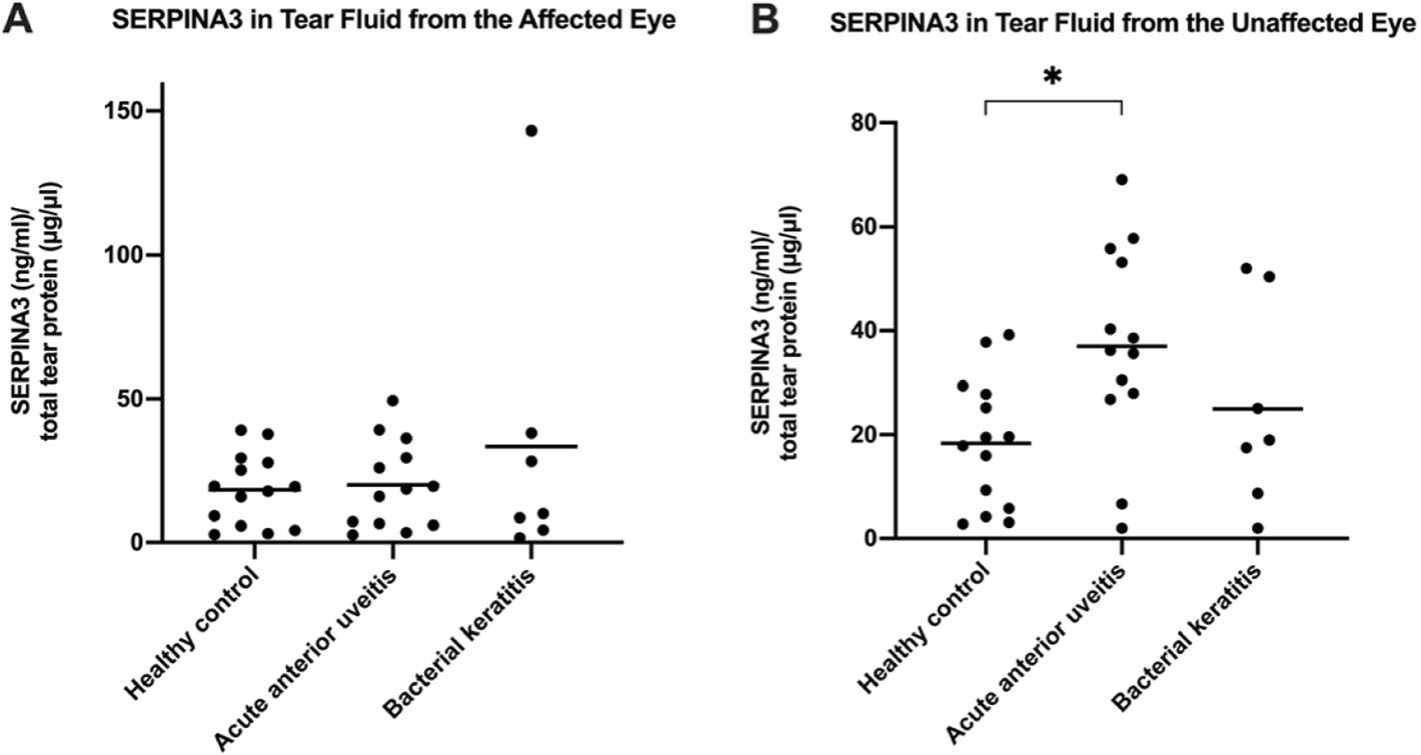
Serpin Family A Member 3 (SERPINA3) was measured in tear fluid of the affected eye (**A**) and unaffected eye (**B**) by enzyme-linked immunosorbent assay in patients with unilateral acute anterior uveitis and bacterial keratitis and in healthy controls. Individual patient values are plotted together with mean values (horizontal lines). *P = 0.019

Serpin Family A Member 3 was chosen for confirmatory ELISA analysis based on its potential biological relevance and results from a prior study by the research group [10]. The proteomic analysis of pooled tear fluid samples in the current study detected an increased level of SERPINA3 in the affected eyes of AAU (fold change: 1.78) and bacterial keratitis patients (fold change: 3.44) compared with the eyes of healthy controls. These results were reflected by the ELISA analysis, which demonstrated a slightly higher, yet non-significant, mean concentration of SERPINA3 in the affected eyes of AAU (20.1 ± 15.2) and bacterial keratitis patients (33.5 ± 50.2) compared with the eyes of healthy controls (18.4 ± 12.4) (Fig. 2a). However, the increased mean SERPINA3 concentration in the affected eyes of bacterial keratitis patients was due to one extreme outlier (Fig. 2a). If this value had been omitted from the analysis, the mean SERPINA3 would have been lower than for healthy controls (data not shown).

The proteomics analysis demonstrated a slightly decreased level of SERPINA3 in the affected eyes relative to the unaffected eyes of AAU patients (fold change: − 1.04). In bacterial keratitis patients, by contrast, the SERPINA3 level was higher in the affected eyes relative to the unaffected eyes (fold change: 2.04). Similar tendencies were seen in the ELISA analysis, in which SERPINA3 was significantly decreased in the affected eyes (20.1 ± 15.2) compared with the unaffected eyes (37.0 ± 19.3; *P* = 0.009) of AAU patients (Fig. 2a and b). In bacterial keratitis patients, the mean SERPINA3 concentration was increased in the affected eyes (33.5 ± 50.2) compared with the unaffected eyes (25.0 ± 19.4) (Fig. 2a and b).

The concentration of SERPINA3 in the unaffected eyes of AAU patients was significantly higher than in the eyes of healthy controls (*P* = 0.019) (Fig. 2b). By contrast, the concentration of SERPINA3 in the unaffected eyes of bacterial keratitis patients did not significantly differ from the eyes of healthy controls (Fig. 2b).

In summary, the ELISA results of the relative abundance of SERPINA3 between affected and unaffected eyes of patients, as well as between patients and healthy controls, corresponded to the proteomics results.

We also compared tear concentration of SERPINA3 in AAU patients that were HLA-B27 positive (*N* = 4) with those who were negative (*N* = 3). There were no significant differences in SERPINA3 concentration when comparing affected eyes of HLA-B27 positive patients (24.5 ± 23.3) with affected eyes of HLA-B27 negative patients (18.6 ± 11.5; *P* = 0.68). Similarly, SERPINA3 concentration in unaffected eyes of HLA-B27 positive patients (42.8 ± 14.0) did not differ significantly from unaffected eyes of HLA-B27 negative patients (37.4 ± 31.2; *P* = 0.76).

**Fig. 3:**
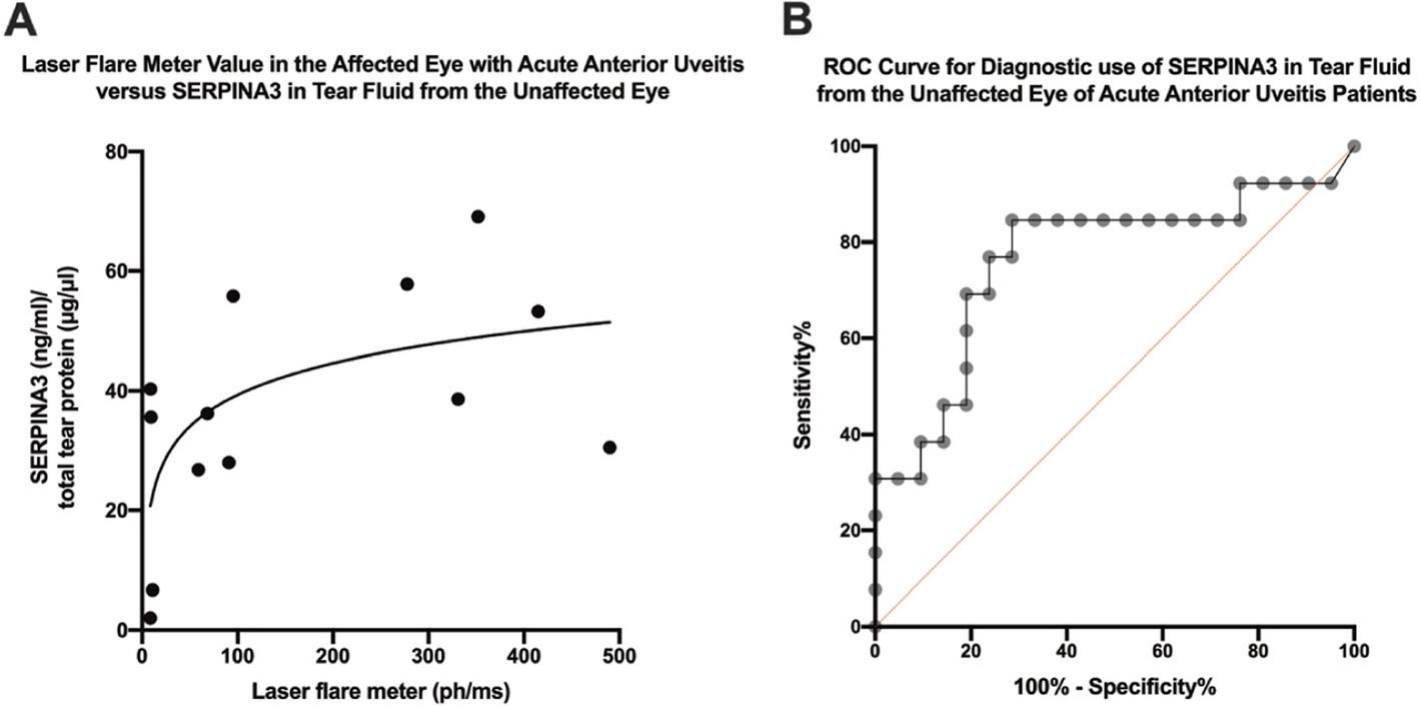
Serpin Family A Member 3 (SERPINA3) was measured by enzyme-linked immunosorbent assay in tear fluid of the unaffected eyes of patients with unilateral acute anterior uveitis and compared with laser flare meter measurements of anterior chamber inflammation of the affected eyes (**A**). Individual patient values are plotted together with the best fitting logarithmic model (curve) (*R*^*2*^ = 0.39; *P* = 0.022). **B** A receiver operating characteristic (ROC) curve was calculated to estimate sensitivity and specificity of varying cutoff values for SERPINA3 concentration for detecting patients with unilateral acute anterior uveitis (area under the curve: 0.764; *P* = 0.011)

We also assessed the relationship between the concentration of SERPINA3 in the unaffected eyes of AAU patients with laser flare meter measurements of the affected eyes of the same patients. Following curve-fitting a significant logarithmic relationship was found; the SERPINA3 concentration appeared to increase relatively rapidly at the lower spectrum of flare values, before leveling off at the higher spectrum (*P* = 0.022) (Fig. 3a). Thus, concentration of SERPINA3 in the unaffected eyes of AAU patients appears to be related to the level of inflammation as measured by laser flare meter in the affected eye.

Since the concentration of SERPINA3 in the unaffected eye of AAU patients was significantly higher than in the eyes of healthy controls, we explored to which extent the measurement of SERPINA3 could be used as a biomarker for unilateral AAU. A receiver operating characteristic (ROC) curve was calculated to estimate sensitivity and specificity of a given cutoff value for SERPINA3 concentration in tear fluid (Fig. 3b). The ROC curve analysis demonstrated an acceptable and significant area under the curve (AUC: 0.764; *P* = 0.011). Optimal sensitivity of 85% and specificity of 71% were obtained by using > 26 as cutoff for SERPINA3 concentration. Consequently, measurement of SERPINA3 in tear fluid can potentially discriminate between AAU and non-AAU patients in the current study.

## Discussion

The current study demonstrated an increased concentration of SERPINA3 by ELISA in tear fluid of the unaffected eyes of patients with unilateral AAU compared with healthy controls. Hence, there is a rationale for exploring the tear fluid proteome of apparently healthy eyes in clinically unilateral conditions. In addition, an inflammatory tear fluid protein profile was shown by proteomics in patients with both AAU and bacterial keratitis.

Patients and healthy control in this study were comparable in terms of age, sex, and ethnicity. A slightly reduced intraocular pressure in AAU patients likely did not affect results significantly. Reduced intraocular pressure in AAU may be the result of either decreased aqueous humor production or increased uveoscleral outflow [28]. Tear secretion, however, varied considerably, being highest in affected eyes of AAU patients. Tear secretion can be classified as basal, stimulated, emotional, and closed-eyes tears [13]. In addition, tear proteins have been termed constitutive (i.e. produced at a constant level), regulated (i.e. their production rate change with level of tear secretion), and serum derived (which decrease whenever tear secretion increases) [9]. For example, in stimulated tear secretion the concentration of the serum protein albumin can decrease to approximately one fifth of the concentration compared to that of unstimulated tear secretion [14]. Other tear proteins, including lactoferrin and lipocalin-1, do not change considerably with the level of tear secretion [13]. Therefore, the type of tear secretion exhibited at the time of tear sampling will affect the composition of tears. In our study, the patients with AAU had more than twice as high Schirmer’s test score compared to the healthy controls. Moreover, the concentration of serum albumin in tear samples from the affected eyes of AAU patients was lower than in the contralateral unaffected eyes and also lower than in eyes of healthy controls. Consequently, for AAU patients, these results could indicate a reduced concentration of serum-derived proteins in tear fluid from the affected eyes due to stimulated tear secretion.

Approximately one third of tear proteins that were increased in the patients relative to the healthy controls were shared between AAU and bacterial keratitis patients. Hence, although both patient populations displayed ocular inflammation, there were considerable differences in which proteins were increased. For AAU patients, the strongest increase in concentration was seen for submaxillary gland androgen regulated protein 3B (SMR3B) and submaxillary gland androgen regulated protein 3A (SMR3A). As SMR3A was increased both compared to eyes of healthy controls and unaffected eyes of patients, this protein was likely produced locally and not serum-derived. The submaxillary gland androgen regulated proteins have been suggested to belong to a new class of anti-inflammatory agents [21]. These proteins have been shown to especially target neutrophils through inhibition of chemotaxis and superoxide production [21]. They have also been demonstrated to protect against LPS-induced shock [20, 21]. Interestingly, our results identified LPS, an inducer of endotoxin-induced uveitis (EIA) [23], as the top upstream regulator in affected eyes of AAU compared with the contralateral unaffected eye.

For patients with bacterial keratitis, the two top upregulated tear proteins were S100 calcium-binding protein A9 (S100A9) and orosomucoid 2 (ORM2), compared with eyes of healthy controls and unaffected eyes of patients, respectively. S100A9 joins S100A8 to form calprotectin, which is highly specific to neutrophils [30]. The quantification of calprotectin in stool is an established biomarker of inflammatory bowel disease, and the protein correlates with the number of neutrophils present in the bowel [24]. Hence, it appears sound that S100A9 is increased in tear fluid of patients with bacterial keratitis. Orosomucoid, also termed alpha-1-acid glycoprotein, is one of the major acute phase proteins in humans, and it exerts immunomodulatory effects [16]. In unilateral Behcet’s disease, alpha-1-acid glycoprotein 1 was increased in tears of the affected eye compared with the contralateral unaffected eye [18]. Orosomucoid is mainly produced by hepatocytes in the liver, but extra-hepatic production sites including leukocytes have also been acknowledged [12]. Hence, ORM2 may have been secreted by local corneal leukocytes; we found high levels of this protein in the affected eyes of patients with bacterial keratitis compared with the unaffected, contralateral eyes.

In accordance with our prior pilot study of AAU patients [10], LXR/RXR activation was among the top canonical pathways activated in AAU patients in the current study. However, because activation of this pathway was also evident in patients with bacterial keratitis, it was not specific for AAU. Furthermore, three of the top five canonical pathways identified were shared between AAU and bacterial keratitis patients.

Serpin Family A Member 3 was increased in the unaffected eyes of patients with unilateral AAU compared with healthy controls. Serpin Family A Member 3 can be induced by interleukin-1 (IL1), IL6, and probably also by the JAK/STAT-pathway [5]. Interleukin-1 and 6 are important cytokines in the pathogenesis of uveitis, and increased levels of these cytokines were found in the aqueous humor of patients with idiopathic and HLA-B27-related anterior uveitis [8]. Expression of SERPINA3 mRNA has been detected in the iris and ciliary body following LPS-injection in rats [27], as well as in a number of other tissues throughout the body [5]. However, our laser flare measurements indicated absence of anterior chamber inflammation in the unaffected eye of the AAU patients, suggesting that local production of SERPINA3 was unlikely. Serpin Family A Member 3 is considered to be produced by the liver and enter the blood stream as an acute phase response protein [5]. Hence, SERPINA3 in the AAU patients most likely represents a primarily serum-derived tear fluid protein. This would explain the lower concentration of SERPINA3 in the affected eyes, which displayed hypersecretion of tears that normally decreases the concentration of serum-derived tear proteins. In a prior study, serum levels of another acute phase protein, alpha-1-acid glycoprotein (also termed ORM), was related to the severity of inflammation in patients with idiopathic AAU [15]. A recent study suggested that the acute phase response protein c-reactive protein (CRP) in serum could be an indicator of the degree of inflammation in anterior uveitis [6]. This finding is in accordance with our study, in which SERPINA3 was correlated with the degree of anterior chamber flare in the affected eyes of AAU patients.

## Conclusions

In conclusion, the current study demonstrated an increased tear fluid concentration of the acute phase response protein SERPINA3 in patients with unilateral AAU compared with healthy controls. To further explore the potential of SERPINA3 as a biomarker, future studies should assess its dynamic properties in tear fluid in active and quiescent uveitis.

## Data Availability

The datasets used and/or analyzed during the current study are available from the corresponding author on reasonable request.

## Abbreviations

AAU: Acute anterior uveitis
ANOVA: Analysis of variance
APP: Amyloid beta precursor protein
AUC: Area under the curve
CRP: C-reactive protein
EIA: Endotoxin-induced uveitis
ELISA: Enzyme-linked immunosorbent assay
FXR/RXR: Farnesoid X receptor/retinoid X receptor
IL1: Interleukin-1
IL6: Interleukin-6
IOP: Intraocular pressure
IPA: Ingenuity Pathways Analysis
JAK/STAT: Janus kinase/signal transducer and activator of transcription
LPS: Lipopolysaccharide
LXR/RXR: Liver X receptor/retinoid X receptor
MAPT: Microtubule associated protein tau
ORM2: Orosomucoid 2
PSEN1: Presenilin 1
ROC: Receiver operating characteristic
S100A8: S100 calcium-binding protein A8
S100A9: S100 calcium-binding protein A9
SERPINA3: Serpin Family A Member 3
SMR3A: Submaxillary gland androgen regulated protein 3A
SMR3B: Submaxillary gland androgen regulated protein 3B
TNF: Tumor necrosis factor
